# Ethnic Differences in Prevalence of General Obesity and Abdominal Obesity among Low-Income Rural Kazakh and Uyghur Adults in Far Western China and Implications in Preventive Public Health

**DOI:** 10.1371/journal.pone.0106723

**Published:** 2014-09-04

**Authors:** Jia He, Shuxia Guo, Jiaming Liu, Mei Zhang, Yusong Ding, Jingyu Zhang, Shugang Li, Shangzhi Xu, Qiang Niu, Heng Guo, Rulin Ma

**Affiliations:** Department of Preventive Medicine, Shihezi University School of Medicine, Shihezi, Xinjiang, China; Old Dominion University, United States of America

## Abstract

**Background:**

The global pandemic of obesity has become a disastrous public health issue that needs urgent attention. Previous studies have concentrated in high-income urban settings and few cover low-income rural settings especially nomadic residents in mountain areas. This study focused on low-income rural and nomadic minority people residing in China’s far west and investigated their prevalence and ethnic differences of obesity.

**Methods:**

A questionnaire-based survey and physical examination of 8,036 individuals were conducted during 2009–2010, using stratified cluster random sampling method in nomadic Kazakhs and rural Uyghur residents (≥18 years old) in 18 villages, Xinjiang, China, about 4,407 km away from capital Beijing. Obesity was defined by BMI and WC.

**Results:**

The overall prevalence of general and abdominal obesity in Kazakh adults were 18.3% and 60.0%, respectively and in Uyghur, 7.6% and 54.5%, respectively. Female’s prevalence of obesity was higher than male’s for general obesity (45–54 age group in Uyghur, P = 0.041) and abdominal obesity (≥55 years in Kazakhs, P_55∼_ = 0.010, P_65∼ = _0.001; and ≥18 years in Uyghurs, P<0.001). Kazakh’s prevalence of obesity was higher than Uyghur’s (general obesity: ≥35 years, P<0.001; abdominal obesity: ≥25 years in males and ≥65 years in females, P<0.01). The prevalence of obesity increased after 18 years old and subsequently decreased after 55 years old. Meat consumption, older age, and female gender had a higher risk of obesity in these two minorities.

**Conclusions:**

Both general and abdominal obesity were common in rural ethnic Kazakhs and Uyghurs. The prevalence rates were different in these two minorities depending on ethnicity, gender, and age. Kazakhs, females and elderly people may be prioritized in prevention of obesity in western China. Because of cost-effectiveness in measuring BMI and WC, we recommend that BMI and WC be integrated into local preventive policies in public health toward screening obesity and related diseases in low-income rural minorities.

## Introduction

Obesity has become a global pandemic affecting 200 million men and nearly 300 million women worldwide posting great public health threats to all nations and all races [1]. According to the World Health Organization (WHO), the worldwide prevalence of obesity (body mass index (BMI) ≥30 kg/m^2^) was almost doubled between 1980 and 2008 with estimated 502 million adults being obese globally by 2008 [2]–[5].

China is a multi-ethnic country and there are more than 10 ethnic groups in Xinjiang Uyghur Autonomous Region, where Kazakhs and Uyghurs are two large inhabitant minority groups. Due to limited resources in public health and poor transportation, there have not been serious investigations to analyze local public health needs including prevalence of obesity and related diseases such as diabetes and cardiovascular diseases. On the other hand, due to differences in religion, culture, lifestyle, diet, and genetic background in these ethnic groups, characterizing the prevalence of obesity may reveal valuable information for making appropriate policies in preventive public health for inhabitant residents in Xinjiang.

In terms of screening for obesity, BMI and waist circumference (WC) measurements are two cost-effective and yet practical indices to identify general and abdominal obesity. It has been reported that in some populations, mean WC increases more rapidly than mean BMI [6]–[10]. It is suggested that using BMI in combination with WC allows more accurate monitoring of obesity.

Studies have shown that prevalence of obesity varies depending on populations and areas [6], [7], [11]–[13]. However, the information is gathered mainly from high-income and urban settings and little is from low-income rural settings. Using BMI and WC, this study has focused on studying two local minority groups, namely Kazakh and Uyghur people, to investigate the prevalence of obesity and possible differences between ethnic groups residing in far western China. Our findings presented may have important implications in preventive public health for medically underserved Muslim minorities.

## Methods

### Ethics Statement

The Institutional Ethics Review Board (IERB) at the First Affiliated Hospital of Shihezi University School of Medicine approved the study (IERB No. SHZ2010LL01). Standard university hospital guidelines including informed consent, voluntary participation, confidentiality, and anonymity were followed. All participants gave written informed consent before the study began.

### Settings and Study Population

The survey was conducted from 2009 to 2010 in Yili (Kazakh) and Kashi (Uyghur) prefectures, respectively, about 4,407 km (2,739 miles) away from Beijing, where approximately 98% of the population are minority Muslim Kazakhs or Uyghurs. Multistage (prefecture-county-township-village) stratified cluster random sampling method was used to select participants. At beginning, we chose these two representative prefectures (Yili and Kashi) according to geographical distributions of the minority populations in Xinjiang, a province in China’s northwest. We randomly selected one county in each prefecture and one township from each county (Nalati Township in Xinyuan County and Jiangbazi Township in Jiashi County). At the last stage, stratified sampling method was used to draw corresponding villages in each township (6 villages in Nalati Township and 12 villages in Jiangbazi Township). We interviewed local Kazakhs and Uyghurs aged 18 years or older residing in the village for at least 6 months. We successfully interviewed a total of 8,036 individuals (3,920 Kazakhs and 4,116 Uyghurs, respectively). The overall response rate was 89.3% (87.1% for Kazakhs and 91.5% for Uyghurs, respectively).

### Questionnaire Survey

A self-developed questionnaire was applied to collect detailed information from all respondents during face-to-face interview. The questionnaire consisted of demographic information of respondents (such as age, gender, ethnicity, education level, and marital status, etc.) and personal lifestyles (such as smoking, alcohol intake, physical activity, and dietary habits, etc.).

### Physical Examinations

Anthropometric measurements of height, weight and WC were obtained using a standard protocol for participants during the interview and physical examinations [14]. Height was measured to the nearest 0.1 centimeter (cm), without shoes, with the participant’s back square against the wall tape, eyes looking straight ahead with a right-angle triangle resting on the scalp and against the wall. Weight was measured with a lever balance to the nearest 0.1 kilogram (kg) in light undergarments without shoes. WC was defined as the midpoint between the lower rib and upper margin of the iliac crest, measured by a ruler tape with an insertion buckle at one end. WC was measured to the nearest 0.1 cm. Body mass index (BMI) was calculated by dividing weight (in kilograms) by height (in meters) squared (kg/m^2^).

### Definitions

To ensure comparability with other studies, our study incorporated the criteria recommended by the Working Group on Obesity in China (WGOC) (General obesity: BMI ≥28 kg/m^2^; Abdominal obesity: WC ≥85 cm for men and ≥80 cm for women) [15]. The WHO classifications for Europids (General obesity: BMI ≥30 kg/m^2^; Abdominal obesity: WC ≥102 cm for men and ≥88 cm for women) [16].

### Statistical Analysis

A databank was created using EpiData software (EpiData Association, Odense, Denmark, http://www.epidata.dk/). Data were then analyzed using SPSS (Statistical Program for Social Sciences, version 17.0, 2008). Continuous variables were presented as means ± standard deviations (M±SD) and analyzed using t-test. Categorical variables were expressed as numbers or percentages and analyzed using the Chi-square test and trend test. Adjusted odds ratio (OR) with associated 95% confidence interval (95% CI) for general obesity, and abdominal obesity were calculated from multivariate logistic regression. The official 2000 census data of China was used to calculate age-standardized rates [17]. All statistical tests were two-sided and differences were considered statistically significant when *P* value <0.05.

## Results

### Basic Characteristics of the Study Populations

Among 3,920 Kazakh adults, there were 1,551 men (39.6%) and 2,369 women (60.4%) with an average age of (44.19±13.24) years. Among 4,116 Uyghur adults, there were 2,036 men (49.5%) and 2,080 women (50.5%) with an average age of (43.56±17.69) years. The average age, BMI, WC and gender differed significantly among ethnicities (*P*<0.001 for each comparison). Kazakh adults had greater BMI and WC compared to corresponding Uyghur adults (**Table 1**).

**Table 1:** Baseline characteristics of the study population.

Characteristic	Kazakh			Uyghur			Total		
	Male	Female	Total	Male	Female	Total	Male	Female	Total
n	1551	2369	3920	2036	2080	4116	3587	4449	8036
Age (years)*	45.10(13.50)	43.60(13.03)	44.19(13.24)	44.17(17.16)	42.96(18.19)	43.56(17.69)	44.57(15.69)	44.30(15.66)	43.87(15.68)
BMI (kg/m^2^)*	24.55(4.14)	24.17(4.35)	24.32(4.27)	22.98(3.12)	22.70(3.66)	22.84(3.40)	23.66(3.68)	23.48(3.10)	23.56(3.92)
WC (cm)*	88.37(12.19)	83.89(12.16)	85.66(12.36)	84.95(12.16)	83.55(25.78)	84.25(19.44)	86.44(10.71)	83.73(19.73)	84.94(16.39)

Note: BMI = body mass index, WC = waist circumference, SD = standard deviation. * = Data were expressed as mean (SD).

### Prevalence of General Obesity and Abdominal Obesity (by WGOC Criteria)

**Table 2** shows the gender- and age-specific prevalence rates of general obesity and abdominal obesity in these two ethnic groups based on the WGOC criteria for BMI and WC classifications. The prevalence of general obesity in Uyghur women was significantly greater than that in Uyghur men in the 45–54 age group (*χ^2^* = 4.156, *P* = 0.041). The prevalence of abdominal obesity in Kazakh women was significantly higher than that in Kazakh men in the 55–64 and ≥65 age groups (*χ^2^*_55−_ = 6.607, *P* = 0.010; *χ^2^*_65−_ = 10.779, *P* = 0.001), respectively. The prevalence of abdominal obesity in Uyghur women was significantly higher than that in Uyghur men in all age groups (≥18 years, *P*<0.001). It was interesting to note that the prevalence of both general and abdominal obesity increased regardless of gender and ethnicity (including Kazakhs and Uyghurs) after 18 years and subsequently decreased after 55 years old. The prevalence of general obesity peaked at 55–64 years of age among Kazakh adults (*χ^2^_male_* = 61.397, *P*<0.001; *χ^2^_female_* = 155.032, *P*<0.001). Among Uyghur adults, the prevalence of general obesity peaked at 35–44 years of age in men and at 45–54 years in women (*χ^2^_male_* = 32.059, *P*<0.001; *χ^2^_female_* = 40.576, *P*<0.001). The prevalence rate of abdominal obesity among Kazakh adults peaked at 55–64 years of age in men and at ≥65 years of age in women (*χ^2^_male_* = 163.483, *P*<0.001; *χ^2^_female_* = 320.470, *P*<0.001). The prevalence of abdominal obesity among Uyghur adults peaked at 45–54 years of age in men and at 55–64 years of age in women (*χ^2^_male_* = 152.084, *P*<0.001; *χ^2^_female_* = 152.988, *P*<0.001).

**Table 2:** Prevalence of general obesity and abdominal obesity in Kazakh and Uyghur rural adults according to gender and age categories^#^–WGOC Criteria^##^.

Age(years)	General obesity						Abdominal obesity					
	Kazakh			Uyghur			Kazakh			Uyghur		
	Male	Female	Total	Male	Female	Total	Male	Female	Total	Male	Female	Total
18–24	1(1.0)	3(1.6)	4(1.4)	4 (1.5)	11 (3.8)	15 (2.7)	15(15.6)	42(22.0)	57(19.9)	55(20.3) *	129 (44.9)	184(33.0)
25–34	26(9.0)	27(6.4)	53(7.4)	25 (5.6)	18 (4.1)	43 (4.8)	122(42.1)	185 (43.7)	307(43.1)	147 (32.7) *	207 (46.7)	354(39.6)
35–44	61 (17.0)	94 (14.6)	155 (15.5)	49 (11.3)	51 (10.0)	100 (10.6)	194(54.2)	361 (56.1)	555(55.4)	227 (52.2)*	371 (72.6)	598(63.2)
45–54	85(22.2)	148 (24.5)	233 (23.6)	29 (9.6)*	51 (14.9)	80 (12.4)	268(70.0)	437 (72.2)	705(71.4)	178 (58.9)*	257 (75.1)	435(67.5)
55–64	88 (28.1)	115 (31.9)	203 (30.1)	19 (6.6)	27 (9.2)	46 (7.9)	229(73.2)*	294 (81.4)	523(77.6)	167 (57.8) *	220 (75.3)	387(66.6)
≥65	25 (22.5)	43 (29.7)	68(26.6)	14 (4.8)	14 (6.8)	28 (5.7)	79(71.2)*	127 (87.6)	206(80.5)	151 (52.2)*	136 (66.3)	287(58.1)
Overall	286 (18.4)	430 (18.2)	716 (18.3)	140 (6.9)	172 (8.3)	312 (7.6)	907(58.5)	1446 (61.0)	2353(60.0)	925 (45.4)	1320 (63.5)	2245(54.5)
*χ2_trend_*	52.40	146.04	193.63	1.70	11.09	10.41	137.98	309.24	437.07	101.08	84.64	168.190
*P* value	<0.001	<0.001	<0.001	0.192	0.001	0.001	<0.001	<0.001	<0.001	<0.001	<0.001	<0.001

Note: ^#^ = Data were expressed as n (%). Descriptive characteristics were compared by χ^2^ test and trend test.

## = General obesity: BMI ≥28 kg/m^2^; Abdominal obesity: WC ≥85 cm for men and ≥80 cm for women.

* = *P*<0.05 versus female from the same ethnic group and age.

### Ethnic Differences in Prevalence for General Obesity and Abdominal Obesity between Kazakh and Uyghur Adults (by WGOC Criteria)

The prevalence rates of general obesity in males, females and overall in Kazakh adults were 14.9%, 15.3%, and 15.0%, respectively and of abdominal obesity, 55.1%, 55.9%, and 53.9%, respectively. The corresponding prevalence rates in Uyghur adults were 6.9%, 7.8%, and 7.4%, respectively for general obesity and 43.7%, 61.3%, and 52.7%, respectively for abdominal obesity. **Table 2** shows that the prevalence rates of general obesity in both male and female Kazakhs were significantly greater than those of corresponding male and female Uyghurs in the age groups of ≥35 years (*P*<0.001 for both comparisons). The prevalence rates of abdominal obesity were the same in Kazakh and Uyghur adults (≥25 years in males, *P*<0.01; ≥65 years in females, *P*<0.01).

### Comparisons of Prevalence Rates of General Obesity and Abdominal Obesity Using WGOC or WHO Criteria

**Figure 1:**
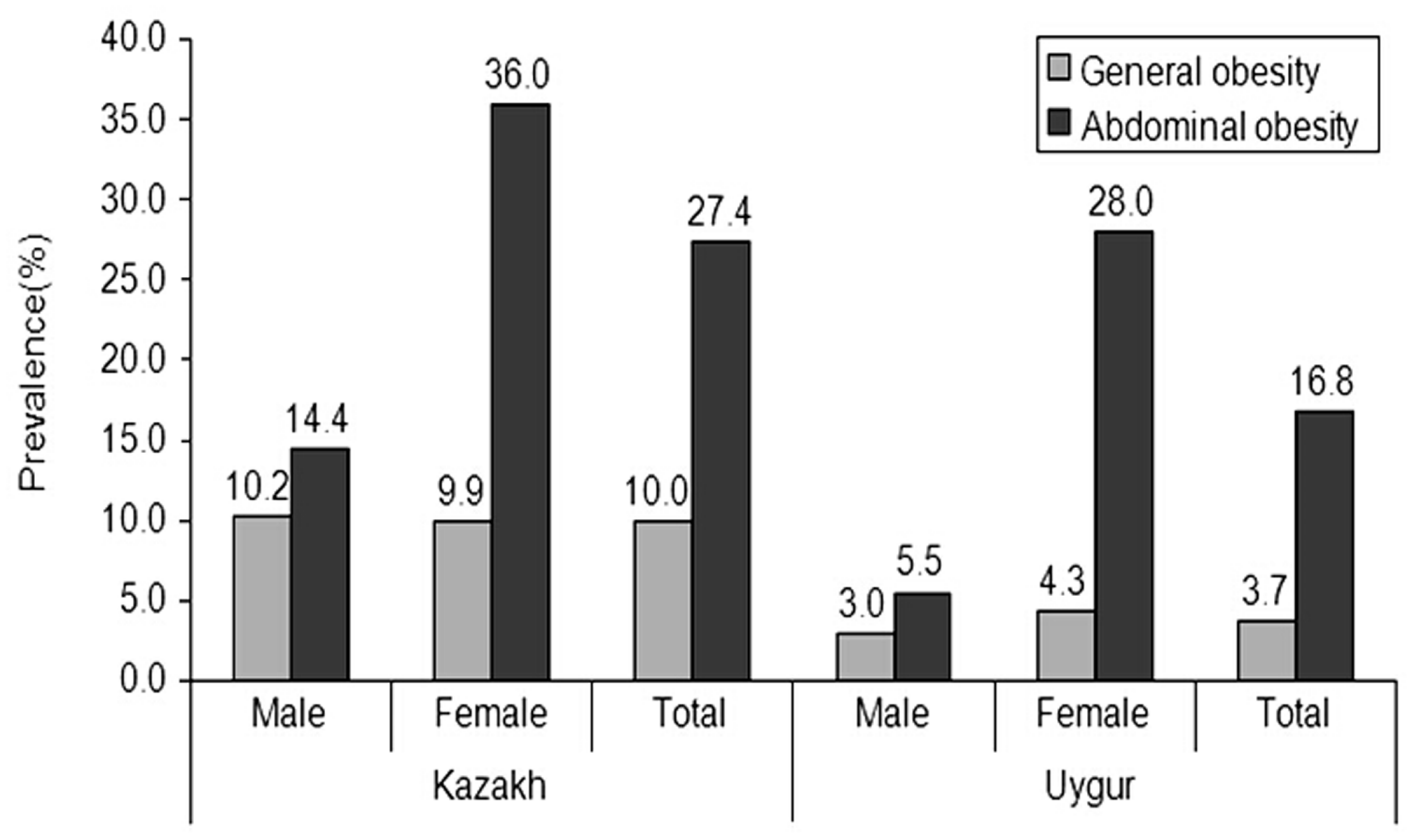
Prevalence of general obesity and abdominal obesity in rural Kazakh and Uyghur adult men and women based on the WHO Criteria. (General obesity: BMI ≥30 kg/m^2^; Abdominal obesity: WC ≥102 cm for men, and ≥88 cm for women).

According to the WHO standards, the prevalence rates of general obesity and abdominal obesity among males, females and both sexes in Kazakh adults were 10.2%, 9.9%, and 10.0%, respectively for general obesity, and 14.4%, 36.0%, and 27.4% respectively for abdominal obesity. The corresponding prevalence rates in Uyghur adults were 3.0%, 4.3%, and 3.7%, respectively for general obesity and 5.5%, 28.0%, and 16.8%, respectively for abdominal obesity (**Figure 1**). For both general and abdominal obesity, the prevalence rates according to the WHO standards were lower than those according to the WGOC standards (*P*<0.001 for either comparison). Further, the prevalence of abdominal obesity was higher than that of general obesity (*P*<0.001 for either comparison).

### Factors Associated with Differential Prevalence of Obesity in Kazakh and Uyghur Rural Adults

As shown in Table 3, we analyzed factors that may be associated with observed differences in prevalence of obesity as defined by BMI-obesity and abdominal obesity (WGOC criteria) in rural Kazakh and Uyghur Adults using multiple logistic regression analysis. Among Kazakhs, meat consumption of ≥2 kg, older age, female gender, smoking, and drinking appeared to render a higher risk of obesity. On the other hand, consumption of 1–3 plates of vegetables was associated with a lowered risk of obesity. In Uyghur adults, however, meat consumption of at 1 kg, older age, female gender were associated with an increased risk of obesity while consumption of 1–3 plates of fruits and higher levels of education received were each associated with a lower prevalence of obesity.

**Table 3:** Factors associated with the difference prevalence of obesity from multivariate logistic regression models in Kazakh and Uyghur rural adults*.

Variables	Kazakh			Uyghur		
	*P* value	*OR* value	*OR 95% CI*	*P* value	*OR* value	*OR 95% CI*
Meat						
No or <1 kg per month	0.004	1.000		<0.001	1.000	
1–2 kg per month	0.721	0.936	0.650∼1.346	<0.001	1.463	1.259∼1.700
≥2 kg per month	0.043	1.467	1.031∼2.124	<0.001	1.621	1.337∼1.965
Sex						
Male	<0.001	1.000		<0.001	1.000	
Female	0.001	1.615	1.214∼2.148	<0.001	2.878	2.479∼3.341
Age						
18–24	<0.001	1.000		<0.001	1.000	
25–34	<0.001	3.422	1.980∼5.916	0.006	1.384	1.098∼1.744
35–44	<0.001	6.342	3.667∼10.970	<0.001	3.818	3.032∼4.808
45–54	<0.001	10.617	5.900∼19.105	<0.001	4.529	3.518∼5.831
55–64	<0.001	10.722	5.662∼20.303	<0.001	4.405	3.404∼5.701
≥65	<0.001	22.726	9.793∼52.739	<0.001	3.425	2.631∼4.459
Vegetables						
No or <1 plate per week	0.108	1.000		**/**	**/**	**/**
1–3 plates per week	0.019	0.327	0.128∼0.834	**/**	**/**	**/**
4–6 plates per week	0.565	0.909	0.658∼1.257	**/**	**/**	**/**
≥7 plates per week	0.276	0.850	0.635∼1.138	**/**	**/**	**/**
Smoking						
No	<0.001	1.000				
Yes	0.010	1.472	1.096∼1.977	**/**	**/**	**/**
Drinking						
No	<0.001	1.000				
Yes	<0.001	2.578	1.601∼4.153	**/**	**/**	**/**
Fruit						
No or <1 plate per week	**/**	**/**	**/**	0.073	1.000	
1–3 plates per week	**/**	**/**	**/**	0.046	0.814	0.665∼0.997
≥4 plates per week	**/**	**/**	**/**	0.058	0.866	0.745∼1.005
Education						
Illiteracy	**/**	**/**	**/**	<0.001	1.000	
Primary school	**/**	**/**	**/**	<0.001	0.606	0.493∼0.746
Junior high school	**/**	**/**	**/**	<0.001	0.350	0.276∼0.443
≥ Senior high school	**/**	**/**	**/**	<0.001	0.374	0.249∼0.562

Note: *OR* = odd ratio, *CI* = confidence interval. / = Data not available. * = Obesity was combined with general obesity and abdominal obesity.

## Discussion and Conclusions

Obesity is a serious chronic disease itself and a significant risk factor for many other diseases such as diabetes and cardiovascular diseases [18]–[19]. Rapid economic growth in China has led to significant changes in life style, including diet and physical activity, which in turn lead to an increase in prevalence of obesity [20]–[22]. Based on the data provided by the China Health and Nutrition Survey (CHNS), the prevalence of general obesity increased 168% from 1993 to 2009 (4.0% in 1993 and 10.7% in 2009) and the prevalence of abdominal obesity increased 101% during the same period (18.6% in 1993 and 37.4% in 2009) [23]. Similar trends in the prevalence of obesity have been observed among US, Spain and Brazil adults [24]–[26]. These observations indicate an urgency to study and prevent this global public health issue of obesity pandemic.

The current study has intended to investigate the epidemiologic features of general and abdominal obesity in rural Kazakh and Uyghur adults in northwest China. Based on WHO criteria, we have found that, in Kazakhs, the overall prevalence rates of general and abdominal obesity in the studied population are as high as 10.0% (general obesity) and 27.4% (abdominal obesity), respectively. In Uyghurs, the prevalence of general obesity has reached 3.7% and that of abdominal obesity, 16.8%, respectively. Using the Chinese WGOC criteria, the corresponding prevalence rates of general obesity in Kazakhs are 18.3%, and abdominal obesity, 60.0%, respectively and the prevalence of general obesity in Uyghurs is 7.6% and abdominal obesity, 54.5%, respectively. As shown in Table 4, the prevalence rates of general and abdominal obesity in rural Chinese Kazakh and Uyghur adults are lower than in urban adults in several developed countries [24]–[26], but are higher than Chinese Han adults no matter in urban or rural areas (general obesity in Uyghur was excepted) [23], [27], especially abdominal obesity. The prevalence rates of general obesity and abdominal obesity in these two minorities are more than doubled as compared with those of majority Han Chinese. Our results of general obesity are consistent with the observations by our colleagues in Kashi Prefecture [28].

**Table 4:** A comparison of general obesity and abdominal obesity from selected ethnic groups within China and in the world.

Ethnicity	Residency	N	Age(years)	GO (%)	AO (%)	Reference
Kazakh	Xinyuan rural, China	3,920	≥18	18.3	60.0	this study*
Uyghur	Jiashi rural, China	4,116	≥18	7.6	54.5	this study*
Han	Urban & rural, China	9,365	≥18	10.7	37.4	[23]*
Kazakh	Xinyuan rural, China	3,920	≥18	10.0	27.4	this study^#^
Uyghur	Jiashi rural, China	4,116	≥18	3.7	16.8	this study^#^
American	Urban, USA	22,872	≥20	33.6	52.9	[24] ^#^
Spanish	Urban, Spain	12,883	≥18	22.9	36.0	[25] ^#^
Brazilian	Urban, Brazil	2,448	≥20	26.1	30.0	[26] ^#^
Han	Urban, China	25,196	18–74	5.2	12.2	[27] ^#^

Note: GO = general obesity, AO = abdominal obesity. * = Using WGOC standard (General obesity: BMI ≥28 kg/m^2^; Abdominal obesity: WC ≥85 cm for men and ≥80 cm for women), **^#^** = Using WHO standard (General obesity: BMI ≥30 kg/m^2^; Abdominal obesity: WC ≥102 cm for men and ≥88 cm for women). Han = Chinese ethnic Han majority.

Gender and age are strongly associated with the prevalence of obesity. Reynolds et al. [29] have reported that women have higher prevalence of obesity than men no matter which cut-offs are used. Wang H et al. [27] have observed that the prevalence of general and abdominal obesity is initially increased and then decreased with age. Our current study has demonstrated similar trends in rural Chinese Kazakh and Uyghur adults, in keeping with previous findings [27]. The gender difference in developing obesity may be due to differences in body composition or sex steroid hormone levels between men and women [30], [31]. Differences in pattern of labor (working outside for men and doing housework for women), levels of physical activity, and social status may also play important roles in the gender difference in developing obesity [32]. Decreased physical activity with age [33], and the survivor effect as those people died at an earlier age due to obesity-related diseases may be closely associated with the subsequent decline in obesity prevalence with age [34]. The gender and age differences in obesity occurrence in rural Chinese Kazakh and Uyghur adults suggest that female and elderly populations should be prioritized in preventive public health in far western China. On the other hand, our findings that females are at a higher risk of obesity may have important implications in national screening programs, such as screening for breast cancer and for cervical cancer currently performed in China, in which a simple addition of measuring BMI and/or WC would give participant women an opportunity for diagnosis of obesity. Furthermore, those women diagnosed as having obesity may be offered chance to be screened for obesity-related diseases such diabetes and cardiovascular diseases.

Our current data show that the prevalence of both general and abdominal obesity in Kazakh adults is higher than Uyghur adults by any definition. Similarly, ethnic differences in the prevalence of obesity have been reported in the US, UK, Norway, and Netherlands [6], [7], [10], [35]–[37]. Kazakhs are nomadic and resides in mountainous areas where outdoor activities are impractical in cold winter season. The primary foods these two minorities consume are wheat, beef, mutton, and dairy products, which contain high fat. Living in plains, Uyghurs drink and smoke less, but consume more fruits and vegetables than Kazakhs. Results by multiple logistic regression analysis let us to suggest that ethnic differences in the prevalence of obesity between Kazakhs and Uyghurs may be due to many factors including age, sex, education, smoking, drinking, vegetables, meat, fruits that are different from other geographic regions in China. On the other hand, molecular studies have demonstrated that Kazakh and Uygur populations reside at borders with countries where Caucasian and Asian peoples are mixed [38]. For example, Uyghurs have a mixture of 60% European ancestry and 40% East Asian ancestry [39], which may explain, at least in part, the genetic influence on the two studied minorities whose abdominal obesity rate is higher than Asians (Han Chinese) but lower than Europeans (Table 4).

Xi B et al. have reported that approximately two-thirds of individuals with obesity may be missed if screening by BMI alone, and WC measurement can provide additional information beyond that provided by BMI for accurate prediction of obesity-associated complications [23]. In this study, the prevalence rate of abdominal obesity is greater than that of general obesity in both rural Kazakh and Uyghur adults may echo those previous findings. Therefore, it is important to use a comprehensive program, including both BMI and WC, to evaluate prevalence, trends, and public health significance of obesity as previously suggested [40]. Furthermore, many studies have suggested that, comparing with Caucasians, Asians have more central distribution of body fat under a given BMI [41], [42], which can magnify the numerical differences between the two types of obesity. Although all types of obesity are significantly increased in Chinese adults, the increase in abdominal obesity is the most significant and such unbalanced increase may further widen the prevalence gap between general obesity and abdominal obesity in Chinese [23]. Moreover, rural adults are more likely to be overweight or obese than urban adults [43], implicating a serious challenge to public health policies in preventing obesity because there would be much limited resources in rural settings especially in rural areas where ethnic minorities reside.

Although this study did not collect social-economic and environmental variables which would have impact on obesity and our study could not establish causal relations due to a cross-sectional design, our study maintained a large sample size in two major minorities in northwest China, which allowed exploring the prevalence of obesity over a range of demographic groups. These findings provide important demographic insights into the growing problem of obesity in rural Kazakh and Uyghur populations.

In both Yili and Kashi Prefectures, most Kazakhs and Uyghurs reside in low-income rural communities where public health resources are limited. For example, in Uyghur concentrated Jiashi county, more than 92% of Uyghurs live on US$1.00 per day or less, a sharp contrast to the national average of 15.9% of people living on US$1.00 per day in 2005 [28], [44]. As chronic diseases such as type 2 diabetes and cardiovascular diseases are common in these low-income rural communities, our data presented here strongly support a cost-effective, two-step screening strategy: first to screen for obesity using the simplest BMI and then to screen persons with obesity for diabetes and cardiovascular diseases using more sophisticated measures [28]. Going one step further to see an immediate impact of this two-step screening strategy on public health, we would like to recommend that BMI and WC examinations be incorporated into the currently running national screening programs for cervical cancer and breast cancer in China. In addition, this two-step screening strategy may also be beneficial in routine health screening in most healthcare organizations by which a large number of obese individuals may be detected and offered preventive measures before they progress to diabetes and/or cardiovascular diseases. In this context, place our observations and recommendations over the making of appropriate policies in public health would not only benefit low-income populations but also help middle-income individuals choose suitable strategies for disease prevention.
